# EEVEE: the Empathy-Enhancing Virtual Evolving Environment

**DOI:** 10.3389/fnhum.2015.00112

**Published:** 2015-03-10

**Authors:** Philip L. Jackson, Pierre-Emmanuel Michon, Erik Geslin, Maxime Carignan, Danny Beaudoin

**Affiliations:** ^1^Faculté des Sciences Sociales, École de Psychologie, Université LavalQuébec, QC, Canada; ^2^Centre for Research in Rehabilitation and Social Integration (CIRRIS), Université LavalQuébec, QC, Canada; ^3^Institut Universitaire en Santé Mentale de Québec (CRIUSMQ), Université LavalQuébec, QC, Canada

**Keywords:** emotions, empathy, pain, FACS, avatar, virtual reality, affective computing

## Abstract

Empathy is a multifaceted emotional and mental faculty that is often found to be affected in a great number of psychopathologies, such as schizophrenia, yet it remains very difficult to measure in an ecological context. The challenge stems partly from the complexity and fluidity of this social process, but also from its covert nature. One powerful tool to enhance experimental control over such dynamic social interactions has been the use of avatars in virtual reality (VR); information about an individual in such an interaction can be collected through the analysis of his or her neurophysiological and behavioral responses. We have developed a unique platform, the Empathy-Enhancing Virtual Evolving Environment (EEVEE), which is built around three main components: (1) different avatars capable of expressing feelings and emotions at various levels based on the Facial Action Coding System (FACS); (2) systems for measuring the physiological responses of the observer (heart and respiration rate, skin conductance, gaze and eye movements, facial expression); and (3) a multimodal interface linking the avatar's behavior to the observer's neurophysiological response. In this article, we provide a detailed description of the components of this innovative platform and validation data from the first phases of development. Our data show that healthy adults can discriminate different negative emotions, including pain, expressed by avatars at varying intensities. We also provide evidence that masking part of an avatar's face (top or bottom half) does not prevent the detection of different levels of pain. This innovative and flexible platform provides a unique tool to study and even modulate empathy in a comprehensive and ecological manner in various populations, notably individuals suffering from neurological or psychiatric disorders.

## Introduction

Imagine a hospital ward for burned people, where a nurse comes in to change the dressings of a patient. As she removes the old dressing, the face of the patient writhes in pain, his body stiffens and he moans. Yet the nurse seems insensitive to his pain and continues her work. This fictional scenario illustrates an extreme situation where healthcare professionals are constantly faced with the pain and suffering of other people, and yet, healthcare professionals are mostly able to function even in this highly discomforting environment. Does this imply that they lack empathy? Surely not, but it suggests that the response that would typically be observed in non-medical personnel, aversion, is somewhat reduced. A number of recent studies in cognitive neuroscience have examined the brain response of people confronted to the pain of others (Jackson et al., 2006; Lamm et al., 2011; Guo et al., 2013) and suggested that observing pain engages brain regions that are also involved in processing painful stimuli, a phenomenon often referred to as “resonance.” Such resonant patterns have been shown to be altered in medical personnel (Cheng et al., 2007; Decety et al., 2010), and also in a number of psychopathologies (Moriguchi et al., 2007; Bird et al., 2010; Marcoux et al., 2014). While this experience-related change in brain response (and in physiological responses, e.g., Hein et al., 2011) can be well-adapted in some contexts, it might also lead in other circumstances to suboptimal interpersonal interactions. For instance, medical personnel should remain empathic, by regulating their own distress without changing their caregiving abilities. In the context of schizophrenia, a reduction of empathy could stem from dysfunctions at different cerebral levels, and could interfere with rehabilitation and therapeutic processes. Thus, having tools to better study the physiological correlates of empathy could lead to new intervention avenues. These examples highlight the need for an innovative tool, which could benefit from the growing literature on the cognitive neuroscience of empathy and the rapid technological advances in both computer animation and measures of neurophysiological responses.

While empathy was initially designated as an ability to “put oneself in the place of another,” as a transposition or mental projection (Dilthey, 1833-1911), this view involves a dissociation between the perception of emotion and cognition (Descartes, 1649). Most contemporary scholars now agree that empathy is the product of both cognitive and emotional processes, and that the line between the two is indeed difficult to draw. Empathy is currently defined as “*The naturally occurring subjective experience of similarity between the feelings expressed by self and others without losing sight of whose feelings belong to whom*” (Decety and Jackson, 2004). The empathy model on which this work is founded is an extension of previously described models, based on multiple fields of research which include developmental, comparative, cognitive and social psychology, as well as psychiatry and neuroscience (Decety and Jackson, 2004; Decety et al., 2007). This model comprises three major components: (1) an automatic and emotional component referred to as *affective sharing* (resonance), (2) a deliberate and controlled cognitive component called *perspective-taking*, and (3) a hybrid component with automatic and controlled processes, dubbed *executive regulation*, which modulates the other components.

One obvious challenge in the study of empathy is that this mental faculty relies largely on inner emotional and cognitive processes, which can only be measured indirectly through largely subjective verbal responses and behavioral measures (Lawrence et al., 2004). Other measures such as psychophysical responses (e.g., skin conductance) and changes in brain activity levels and patterns [e.g., as measured with functional magnetic resonance imaging (fMRI) or electroencephalography (EEG)] have been identified as potential markers of the empathic response (Wicker et al., 2003; Jackson et al., 2005; Marcoux et al., 2013) and have the advantage of being objective. Studies combining several of these markers are scarce, yet the convergence of multiple sources of information seems to be the most promising route to grasp the full complexity of empathy. Most studies still use relatively simple visual stimuli and experiments based on a series of independent short events, in which there is no feedback to the participant. Participants in these studies observe a situation and are asked to rate it on different features, but rarely does the situation change according to the participant's response. Such a form of interactivity seems essential to the ecological study of empathy (Zaki and Ochsner, 2012; Achim et al., 2013; Schilbach et al., 2013). One way to improve this interactive factor in an experimental setting would be to alter the “other,” i.e., the person with which one should empathize, in real time to provide feedback. Research in computer science, through developments in virtual reality, social gaming and affective computing research, is making progress toward improved interactivity in experimental settings (e.g., Vasilakos and Lisetti, 2010; Tsai et al., 2012; de Melo et al., 2014; Gaffary et al., 2014). Affective computing research aims to develop software that can better recognize and display emotions. This type of technology can now be used in combination with the objective neurophysiological markers of human empathy to pinpoint the most relevant and help study online changes in these markers during social interactions.

The systematic study of empathy is limited by the fact that this process may change based on context and on the individuals present in the interaction. Indeed, the literature suggests that empathic responses are different depending on the nature of the relationship between the observer and the target (Monin et al., 2010a,b). Thus, studying empathy systematically requires flexibility both in the choice of markers and in the level of experimental control over the context.

The aim of this article is to describe the recent methodological progress arising from the development of a uniquely powerful interactive virtual reality platform for empathy research. This platform, EEVEE, the Empathy-Enhancing Virtual Evolving Environment, was developed in order to better understand the different behavioral and physiological markers of human empathy. EEVEE was designed based on three objectives: (1) to provide a means to study empathy within an interactive, yet controlled, social environment, (2) to identify the biomarkers that reflect the different facets of the empathic response, and ultimately, (3) to use this technology to train and improve empathy. EEVEE uses avatars that dynamically respond to the user's physiological feedback through the production of emotional facial expressions. At this stage of development, EEVEE is geared toward expressions of pain, as they have been a good model for the study of empathy from a social and cognitive neuroscience perspective (Decety and Jackson, 2004; Coll et al., 2011; Lamm et al., 2011). The use of avatars instead of pre-recorded videos of facial expressions has the advantage of being highly controllable. EEVEE can independently change several features of the facial expression, thereby producing different combinations of expressions of varying (and measurable) intensity, duration, and context. EEVEE can also alter these features in real time based on individual responses, i.e., behavioral, neural and physiological markers. EEVEE has a modular architecture enabling additional instruments and types of neurophysiological measures to be added as they become available.

The first part of this article describes EEVEE and its different components. Then, a two-stage validation process is described, in which the properties of different facial expressions of emotions are demonstrated. A sample experiment follows, showing how EEVEE can be used to test specific hypotheses regarding pain and empathy. Finally, we discuss and provide examples of how EEVEE can be used in real time.

## Development of EEVEE

**Figure 1:**
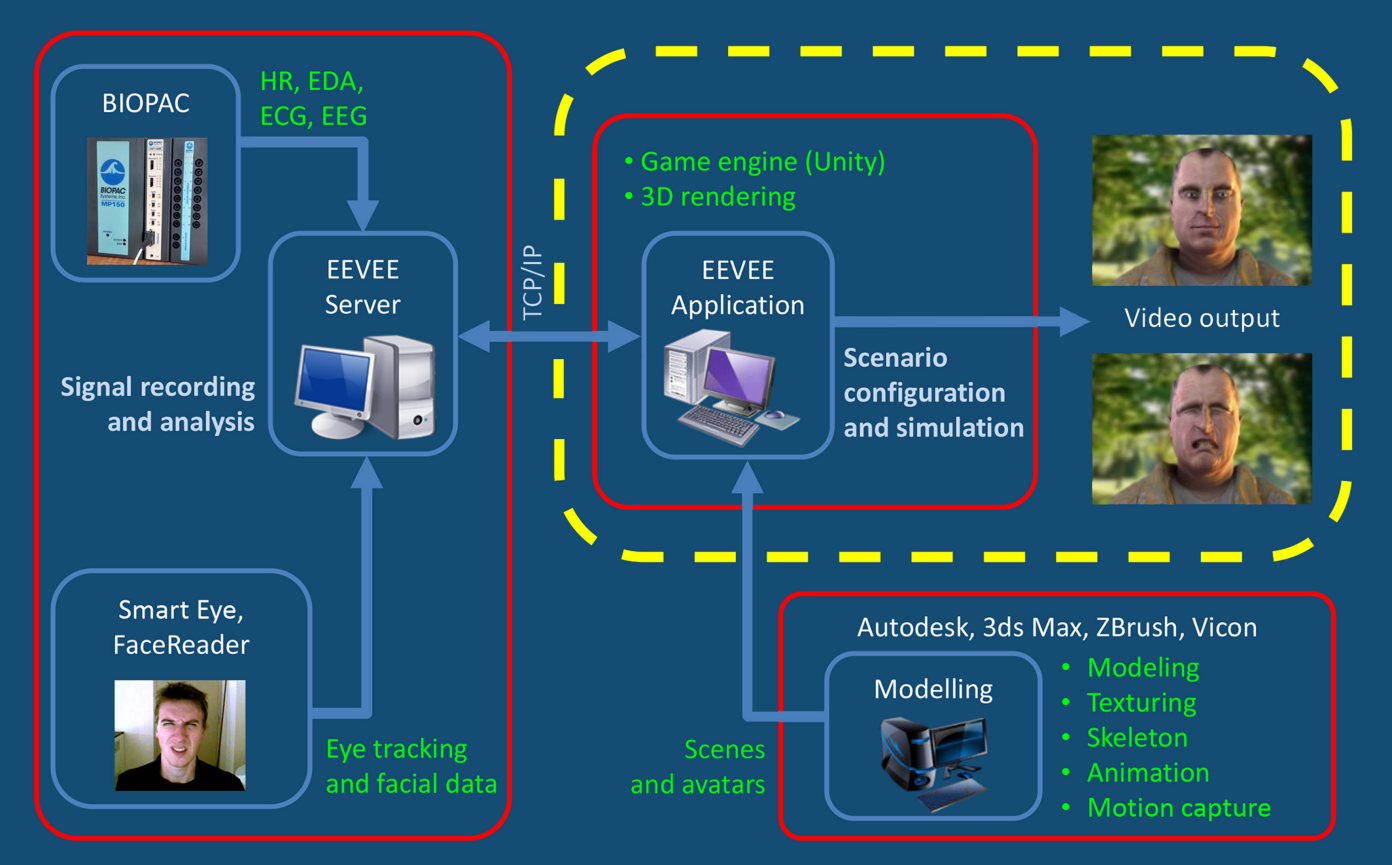
**This figure shows that the EEVEE platform works on the basis of an iterative loop between the observer and the avatar.** The two protagonists, real and virtual, interact in this paradigm through expressions of their emotions. EEVEE project allows implementing new physiological measures functions depending of their relevance and availability. Currently, EEVEE focuses on Central Nervous System (CNS) and Autonomic Nervous System (ANS) responses via heart rate Beat per Minute (BPM), Electrocardiography (ECG), respiration (RESP), Electro-Dermal Activity (EDA), and Automatic Facial Expression Recognition (FACS). The sampling frequency depends on the physiological signal being processed, and varies from 10 Hz (BPM) to 2000 Hz (EEG). The main markers that are extracted include heart beat acceleration and deceleration (BPM), RR interval and their standard deviation (ECG), respiration acceleration, deceleration and apnea (RESP), skin conductance level and skin conductance response's amplitude and area under the curve (EDA), facial action unit intensities (FACS). All of these data are gathered by a system for physiological measurement (MP150, Biopac Systems Inc.) and the FaceReader™ software encodes FACS information.

EEVEE consists of three main components (see Figure 1): (1) the production, animation and display of responsive human avatars, (2) the measure of neurophysiological and motor responses, and (3) a multimodal interface for setting the parameters of the computational integration and of the interaction between avatar and participant within a scenario.

### Production, animation and display of responsive human avatars

**Figure 2:**
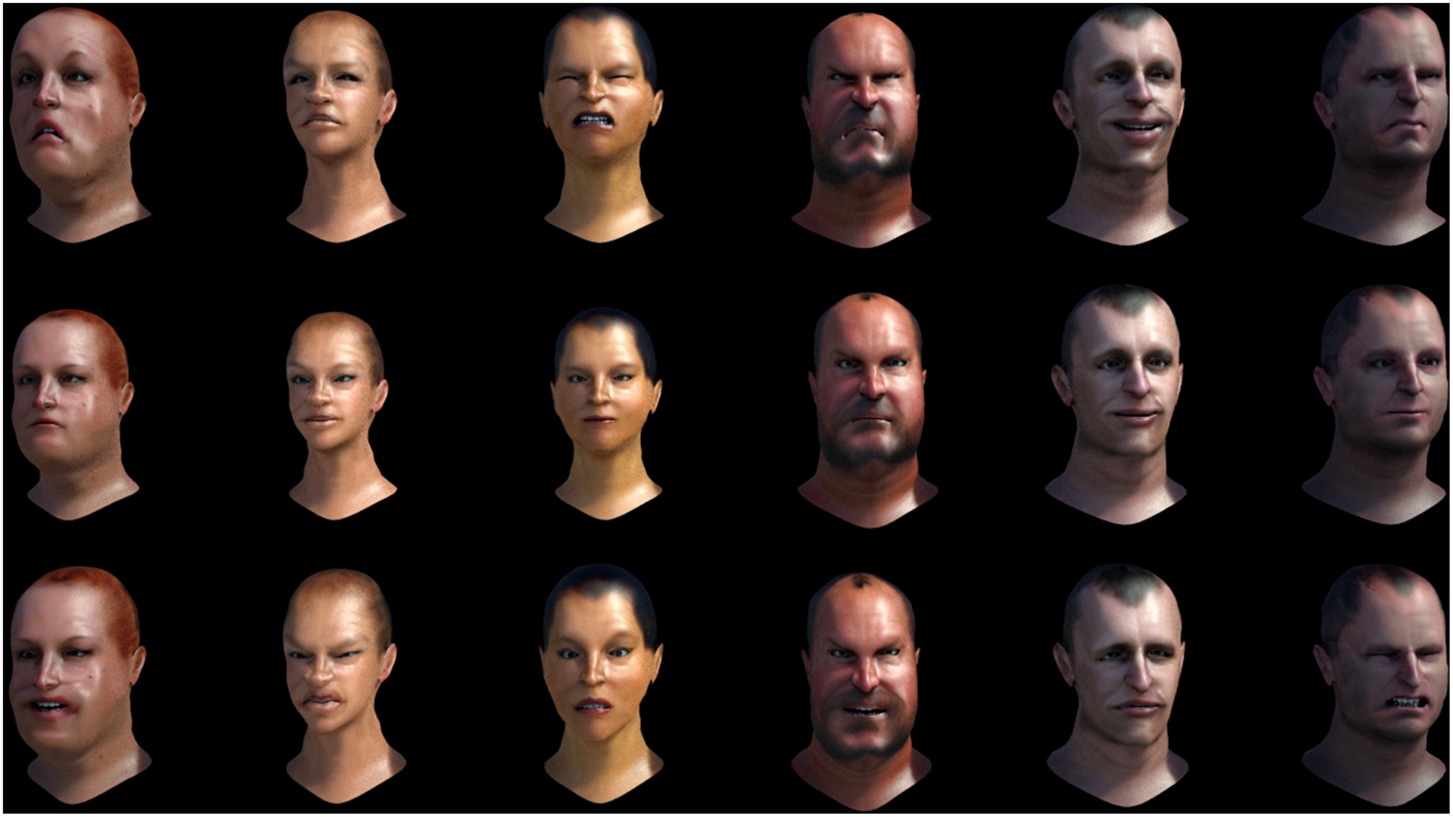
**Examples of avatars' emotional expression of emotions using the Facial Action Coding System principles. Top row** (left to right): Fear, Sadness, Pain, Anger, Joy, Disgust. **Middle row**: Neutral. **Bottom row** (left to right): Joy, Disgust, Fear, Anger, Sadness, Pain.

This component allows the production and animation of high-resolution avatars to produce distinct sets of emotional expressions based on the Facial Action Coding System (FACS), which encompasses 45 facial muscle movements and 10 head movements (Ekman et al., 2002; for pain, see Prkachin and Solomon, 2008). Six different avatars (male and female adults) can currently be selected (see Figure 2). Future versions of EEVEE will allow users to change the avatar's gender, age, and ethnicity independently.

**Figure 3:**
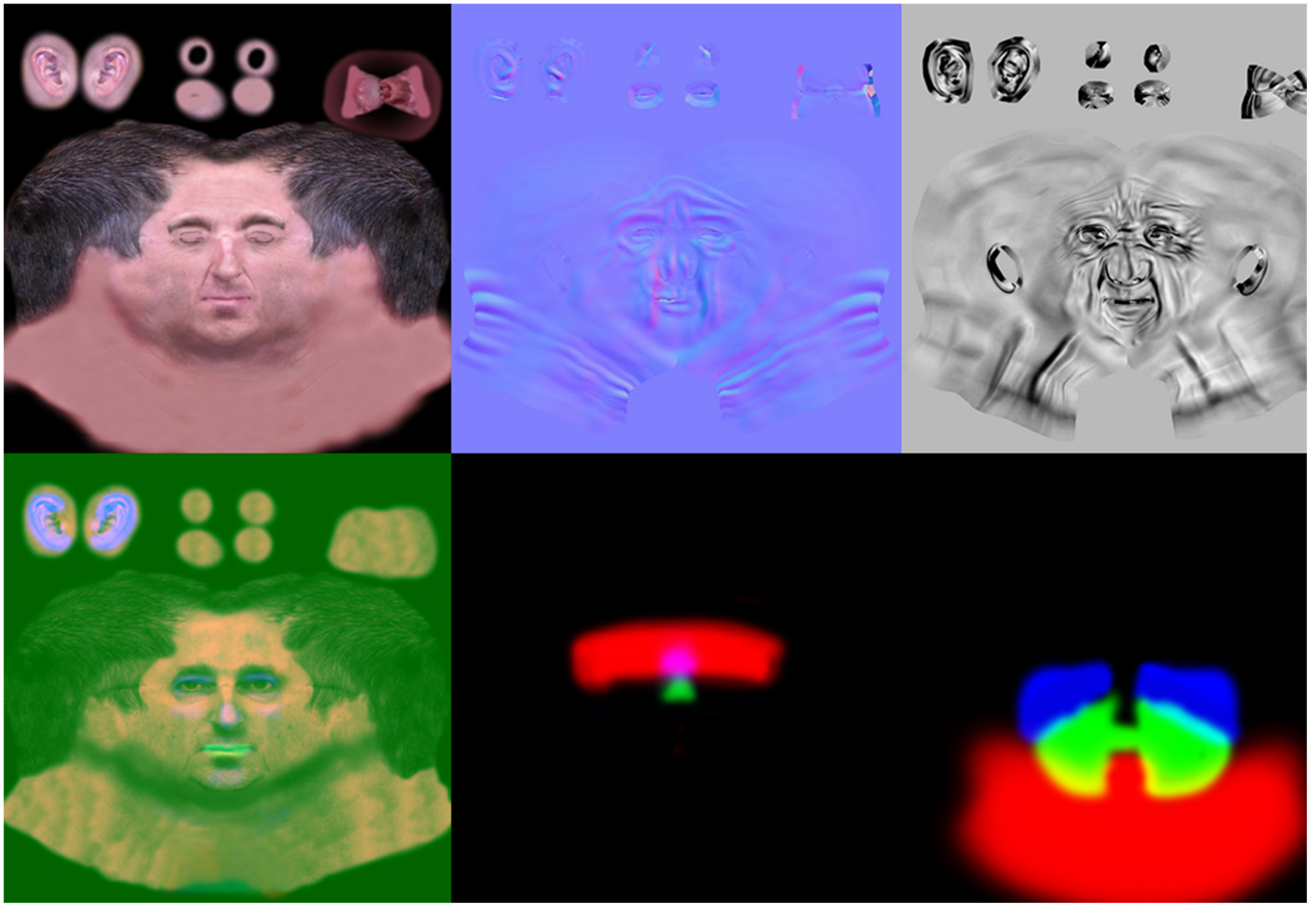
**Dynamic normal maps generated for animating avatars using the Facial Action Coding System principles**.

We implemented a methodology for scanning the head of a real person using a high-resolution 3D scanner (Creaform Gemini™ with the Geomagic Wrap® software). Once scanned, these high-resolution 3D models are transferred by wrap iterations on a single basic low polygon model. This model is able to express a variety of emotions by the use of motion captures (to simulate natural movements of intermediate idle phases between expressions), and blend shapes. Blend shapes (morph target animations) are convex combinations of *n* base vectors and mesh formed in the same topology. The movements of vertex points in xyz space generate animations of shapes. In this first stage of development, on the basis of the 3D model of a team member head, we used 3ds Max® 2014 (Autodesk) to recreate a low-polygon mesh model for real-time 3D use. Based on this, we created a highly detailed and triangulated model in ZBrush® (Pixologic). This high-resolution model was then used to generate a projection texture normal map (Sander et al., 2001), simulating facial detail in the Unity 3D engine (Unity Technologies). The skin of the avatars was produced by combining a Microsoft DirectX® 11 Shader Model 5, using a diffuse texture of 4096 × 4096 pixels based on RGB 173.100.68, and texture normal map of 4096 × 4096 pixels integrating wrinkles to the expressions. A third 4096 × 4096 map contains full texture in each RGB channel: three textures refer to levels of specular, glossiness and depth sub surface scattering (SSS) defining skin translucence. EEVEE display support includes tessellations with displacement map and Phong smoothing, diffuse scattering with separate weighted normal, Fresnel specular reflectance, translucency, rim lighting and real time shadows from two light sources (see Figure 3). Tessellation simulates a mesh of a large volume on a low-polygon mesh, which results in the facial shape of the avatar appearing less angular. Specular reflectance and translucence simulate various aspects of skin gloss, grain, depth and light absorption. Based on the modeling of a first avatar generated with blend shapes, five other avatars were created with very different physical characteristics, but all having the same basic triangulation meshes with 5126 triangles. Importantly, this technique allows the same blend shapes to be used for the expressions of different avatars, while maintaining their idiosyncratic aspect based on their own morphology. We also used a skinning face of the basic avatar, on a bone network for the application of facial motion captures made with a Vicon Bonita™ B10 system, using a 1.0 megapixel camera at 250 frames per second. This skinning face allows the use of motion captures combined with changes in vertex blend shapes. The avatars display special wrinkles only when expressing emotions. Action units (AUs) were created by modifying the vertex positions in our main avatar by a developer with a FACS Coder certification. These blend shapes were then exported to the Unity 3D engine to be dynamically used on all avatars. The blend shapes produce muscular facial movements, based on the muscular movement anatomy described in the FACS Manual (Ekman and Friesen, 1978), and use dynamic normal maps to generate wrinkles associated with the expression of emotions. These wrinkles were created in ZBrush® from a 2048 × 2048 pixel normal map texture, based on Ekman's FACS. The intensity mapping for maximum intensity (E) was determined with a top-down approach using the FACS manual, interpreted for the bone and muscle structure of the human face used as a model in the original avatar. Expression levels Neutral to E were linearly distributed between a completely relaxed state (0%) and maximum expression intensity (100%). The dynamic normal maps display the blending of each of the two RGBa textures that produces four different masks.

**Figure 4:**
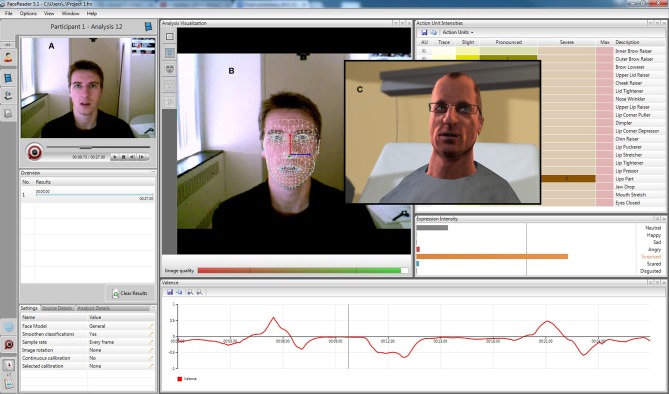
**EEVEE mirroring mode. (A)** Raw live video feed, **(B)** mesh analysis and expression intensity levels from the FaceReader™ software, and **(C)** avatar mirroring of the facial expression generated by EEVEE.

In this first phase of development, we created two different environments within EEVEE: a hospital room (see Figure 4) and a park (see Video 1 in Supplementary Material). These environments can easily be changed to other settings, depending on the user's objectives. They are entirely in 3D, are real-time ready, and can be displayed through the Unity engine on a variety of screens, or even in a more immersive display system such as a HMD (head-mounted display) or a CAVE (computer assisted virtual environment). A version of EEVEE was developed for the Oculus Rift™ HMD (Oculus VR), which improves visual immersion, but also comes with other restrictions, notably for the possibility of using eye-tracking systems.

### Pain facial actions units

**Figure 5:**
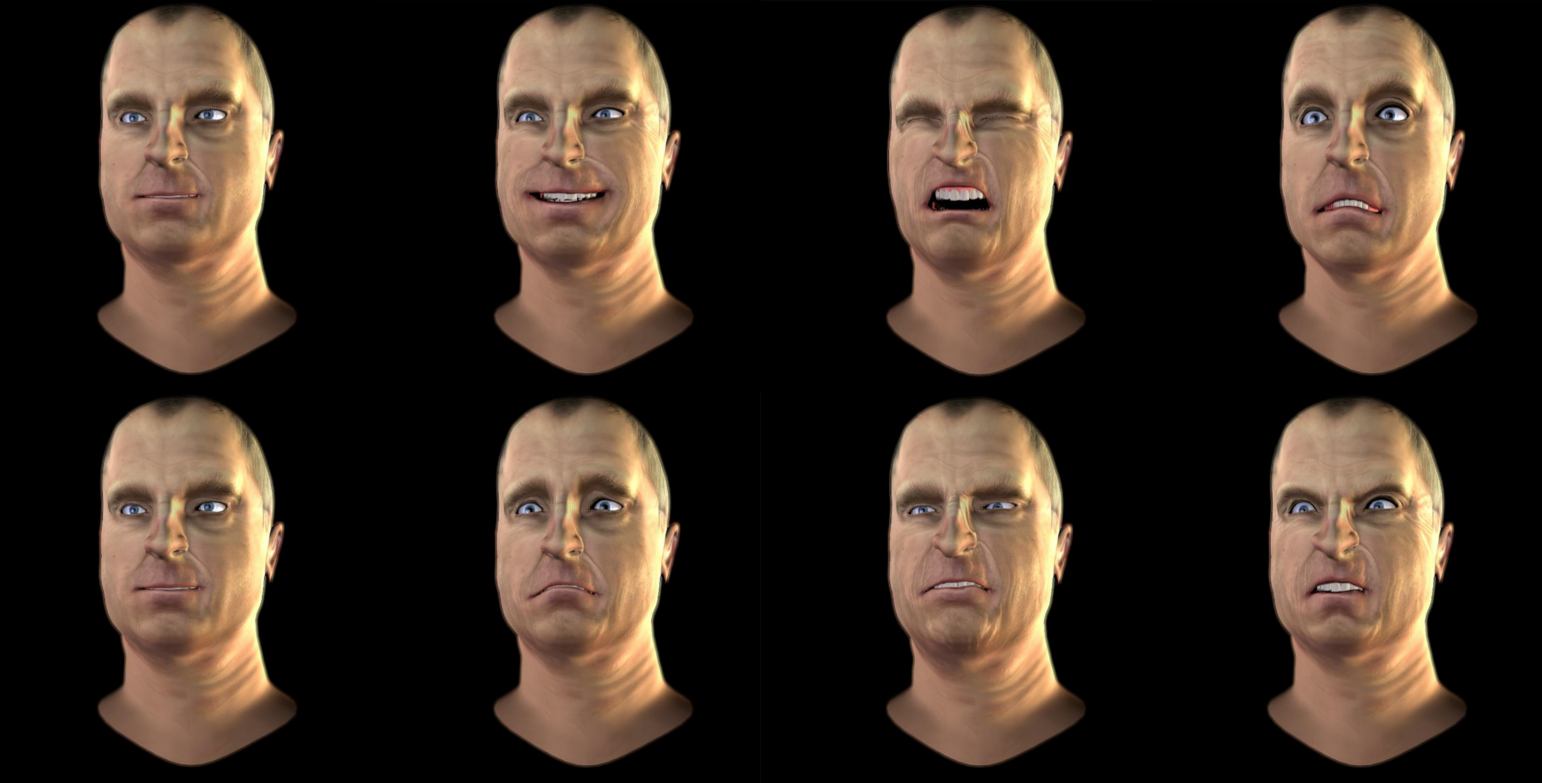
**Facial expressions of emotions displayed by one of the male avatars used in Experiment 1a. Top row** (left to right): Neutral, Joy, Pain, Sadness. **Bottom row** (left to right): Neutral, Fear, Disgust, Anger.

The flexibility of the emotional expressions in EEVEE is a fundamental part of its development. The facial expressions of the avatars are based on the Facial Action Coding System (FACS; Ekman and Friesen, 1978), which is based in part on earlier research by Carl-Herman Hjortsjö on facial imitation (Hjortsjö, 1969). Contractions and relaxations of the different facial muscles create variations that are encoded as Actions Units (AUs). The FACS describes 46 numbered AUs, each AU corresponding to the contraction and relaxation of a muscle or muscle group. In the case of pain for example, several AUs are mobilized: AU4 + (AU6, AU7) + (AU9, AU10) + AU43 (Prkachin, 1992, 2009; Lucey et al., 2011a). AU4 corresponds to the brow lowerer, glabellae, depressor supercilii, corrugator supercilii; AU6 to the cheek raiser, orbicularis oculi (pars orbitalis); AU7 to the lid tightener, orbicularis oculi (pars palpebralis); AU9 to the nose wrinkler, levator labii superioris alaeque nasi; AU10 to the upper lip raiser, levator labii superioris, caput infraorbitalis, AU12 to the lip corner puller, zygomaticus major; AU25 to lips part, depressor labii inferioris, or relaxation of mentalis or orbicularis oris; and AU43 to the eyes closing, relaxation of levator palpebrae superioris. The AUs can be scored according to their intensity by appending letters A–E (from minimal to maximal intensity) following this scale: A'= trace; B = slight; C = marked or pronounced; D = severe or extreme; E = maximum. Using this system, a face with the FACS values AU4B + AU6E + AU7D + AU9C + AU10D + AU12D+ AU25E + AU43A, would result in a pain expression. We also use the units AU51 (head turn left), M60 (head-shake side to side), and M83 (head upward and to the side), to produce natural head movements of the avatars (see Figures 2, 5). The timing of the different AUs is currently the same, but further developments of the system are expected to introduce variable timing of the different AUs of one emotion, which should help approximate the natural dynamics of facial expression (Jack et al., 2014), as well as allowing the study of the impact of changing inter-AU timing.

According to the FACS Manual, wrinkles are often one the first indication of muscular contractions (Ekman et al., 2002). For most AUs, wrinkles appear at the B intensity level. In order to respect this natural dynamic, and in order to avoid the so-called “Uncanny Valley” (Mori, 1970; Seyama and Nagayama, 2007) due to cognitive incoherence during observation of emotional expressions (wrinkles produced by the normal map would be seen very late after users cognitively detected the movements produced by the vertex blend shapes), blend shapes (BS) and dynamic normal maps (DNM) were created following this rule: AU-A: 20% BS + 20% DNM; AU-B: 40% BS + 60% DNM; AU-C: 60% BS + 80% DNM; AU-D: 80% BS + 100% DNM; AU-E: 100% BS + 100% DNM.

### Measures of neurophysiological and motor responses

This component of EEVEE consists of a series of apparatus that will allow real time measurement of behavioral and physiological responses of the participants. A number of inputs have already been integrated in EEVEE, and the number and types of devices can be changed and optimized over time. The current version of EEVEE uses an emotional face recognition tool (Noldus FaceReader™), measures of heart electrical activity, respiration rate, and skin conductance (MP150, Biopac Systems Inc.), as well as eye-tracking and pupillometry (Smart Eye Pro, Smart Eye®).

The instant values for 19 of the FACS AUs and aggregated values for 6 mood expressions detected on the observed face are acquired using Noldus FaceReader™ with the action units and external data modules. This information is transmitted by TCP protocol at 15 Hz. The MP150, Biopac Systems Inc. system is used to acquire: electrocardiographic activity recorded with 3 AgCl electrodes with 10% saline gel positioned using Einthowen's triangle; thoracic dilatation recorded by a stretch mesh strap and transducer; and skin conductance recorded by 2 AgCl electrodes with 0.5% isotonic gel positioned on the index and middle fingertips. These three signals are sent by TCP protocol at a 120 Hz sampling rate, then transformed respectively into heart rate variability measure (difference between the last R-R interval and its previous value), respiration rate variability (difference between the last respiratory cycle duration and its previous value), and galvanic skin response (area under the curve for the last 4 s of a high pass 0.05 Hz filter of the skin conductance). Smart Eye Pro, Smart Eye® is used to acquire eye tracking data and pupil diameter for both eyes at 120 Hz through TCP protocol, with the latter transformed into a pupil diameter variation during the last second. All the physiological data arrive independently to a dedicated server.

### Multimodal interface and online computational integration

This component of EEVEE translates the different responses (behavioral, physiological, neurophysiological) into vectors that can modulate the motor responses of the avatars (facial expression, eye/head movements) thereby producing a truly interactive task. The signal processing, feature extraction and scenario for the avatars' responses are configurable through a scenario designer implemented in a standalone EEVEE application. All of these parameters are being built into a modifiable, simple, and intuitive drag-and-drop interface. It is also possible to program more complex scenarios, thus enabling a great variety of contexts. The system is currently designed to project one avatar at a time, but the projection of two avatars or more will be part of the next development phase. Depending on the interaction mode selected, the avatar's animation (facial features and posture) can be predetermined or triggered in real time to respond to the observer's behavior, posture or physiology. It is also possible to activate a mirror mode for the avatar: recognizing and imitating the facial expressions of the user (see description of the modes below; see Video 1 in the Supplementary Material for a demo of the mirror mode). A dedicated EEVEE server is used to collect, over the network, all the data acquired through different application program interfaces (APIs) and software development kits (SDKs), making it possible to display the avatar and collect the physiological measures at different sites. The signals are then processed with mathematical routines to extract their salient features and reduce their bulk. Such signal processing includes Fourier transforms, low and high pass filters, smoothing, interpolation, etc.

EEVEE can be used in different modes. In the Offline mode, the animations for emotional behavior can be set in advance in the scenario along with its time course and the multiple cues (responses) from an observer watching the avatar can be recorded for post-experiment analysis. In the Mirror mode, EEVEE can be used to reproduce the movements and facial expressions of the observer (by mimicking the AUs detected). The Real-time EEVEE mode will use different combinations of the observer's responses to guide the avatar's behavior (facial expression, posture, eye activity, animation) and create an interactive exchange based on information not typically accessible to people.

### Validation of EEVEE

In order to validate the capacity for EEVEE's avatars to convey specific emotional content through facial expressions, two experiments were conducted. A first two-step experiment was run in which participants had to evaluate the avatars' expressions. A second experiment was conducted in order to test whether participants could assign the target emotion, in this case pain, to avatars whose face was partly masked. This latter experiment also included facial expressions of emotions produced by real actors, which allowed the comparison of pain detection in humans and avatars.

### Experiment 1: validation of the avatars' expressions

#### General objective

Our first experiment was twofold: Experiment 1a (negative emotion discrimination) consisted in asking healthy adults to discriminate between negative emotions depicted by four different avatars (2 males; 2 females) displaying dynamic emotional expressions; Experiment 1b (pain level discrimination) consisted in asking the same group of adults to specifically evaluate pain intensity in different pain expressions from the same avatars.

#### Experiment 1a: Negative emotion discrimination

Our goal in Experiment 1a was to determine whether people could discriminate between four negative emotions (pain, disgust, anger, and fear) depicted by avatars. Pain was the main emotion of interest, but we used fear, anger and disgust as distractors with similar negative valence. These other expressions have the advantage of containing part of the same set of action units (AUs) as facial expressions of pain, such as nose wrinkler, upper lip raiser, brow lowerer, and upper lid raiser (Kappesser and Williams, 2002; Simon et al., 2008; see Table 1).

**Table 1:** **Action Units (AUs) typically recruited when expressing fear, anger, disgust and pain, according to the Facial Action Coding System (FACS)**.

**Action Unit**	**Fear**	**Anger**	**Disgust**	**Pain**
Inner brow raiser (AU1)	X			
Outer brow raiser (AU2)	X			
Brow lowerer (AU4)	X	X		X
Upper lid raiser (AU5)	X	X		
Cheek raiser (AU6)				X
Lid tightener (AU7)	X	X		X
Nose wrinkler (AU9)			X	X
Upper lip raiser (AU10)				X
Lip corner depressor (AU15)			X	
Lower lip depressor (AU16)			X	
Lip stretcher (AU20)	X			
Lip tightener (AU23)		X		
Jaw drop (AU26)	X			
Eyes closed (AU43)				X
^*^Total AUs	7	4	3	6

* *Note that this list is inclusive and that there are individual differences in the degree to which each AU is involved*.

We expected that the participants would differentiate the expressions by attributing more intensity to the target emotions. We also expected that most expressions would lead to the attribution of more than one emotion, as emotions are less prototypical than is often believed (see for instance: Du et al., 2014; Roy et al., unpublished manuscript).

#### Methods

***Participants***. The participants in this study were 19 adults (10 women) recruited through advertisement on the campus of Université Laval. They were aged between 20 and 32 years (*M* = 22.6 years; *SD* = 2.93 years). Exclusion criteria consisted in having a neurological or psychiatric disorder, a medical condition causing pain, working in healthcare or with people suffering from painful conditions, or having previously participated in a study on pain expressions. The study was approved by the Research Ethics Committee of the Institut de réadaptation en déficience physique de Québec. Written informed consent was obtained from all participants and they received 10$ for their participation.

***Material/Task***. Participants were presented video clips of four different avatars showing dynamic facial expressions. The clips displayed the upper body, from the shoulders up, of avatars facing the camera at a 5–10° angle (see Figures 4, 5), dressed neutrally, without hats or accessories. Each clip lasted 3 s, and displayed either neutral expressions or one of the following four negative emotions: pain, disgust, anger and fear. Each emotion was shown at 5 levels (A, B, C, D, and E of the FACS; Ekman and Friesen, 1978), and the neutral clip showed no facial contraction for the whole 3-s clip. In each non-neutral clip, the expression (AUs levels) linearly increased for 2 s from a relaxed state (neutral FACS) to reach the target expression level (either A, B, C, D, or E), which was maintained for 1 s (see examples in Supplementary Material Videos 2–6).

***Procedure***. During the experiment, which lasted about 45 min, participants were gathered in small groups of 1–8 individuals in the front rows of a classroom and were asked to rate a series of video clips displayed on a large screen (175 × 175 cm) placed at the front of the classroom, 3–5 m from the participants. They were asked to rate each video on answer sheets that provided four Visual Analogue Scales (VAS) for each video. After a short tutorial block of 8 trials to familiarize participants with the clip presentation pace and the rating scales, they were presented with four blocks of 21 trials. Within each block (lasting about 7 min), the order of presentation was pseudo-randomized (i.e., constrained to avoid the repetition of three successive clips of the same gender, emotion or level). During each trial, the 3-s clip was repeated 4 times, with an inter-stimulus interval (ISI) of 500 ms. In order to identify the emotion or set of emotions they thought was expressed in each clip, participants were instructed to rate the intensity of each of four emotions displayed by the avatar by making small vertical marks on four separate VAS labeled respectively “Anger,” “Disgust,” “Pain,” and “Fear.” The order of the scales within subject was kept constant for each item during the experiment. The order was also the same across participants. This order could have been varied across subjects to avoid potential order effect from the list, but the clips themselves were randomized and this is where an order effect would be most probable, if any. Each VAS was 10 cm long, with anchors labeled 1 (extreme left) and 100 (extreme right). Participants were explicitly instructed to leave the VAS blank if they thought one emotion was not expressed in the clip, and leave all VAS blank if they did not detect any of the four target emotions. For each VAS, the distance from the left end of the line to the mark made by the participant was measured in millimeters to provide an intensity score (out of 100), with any blank scale attributed a score of 0. A composite score of Total Intensity was computed for each item by adding the four scores provided for this item. An accuracy score was computed for each item as the ratio of intensity for the target emotion scale divided by the Total Intensity score for this item. Additionally, a binary concordance score was computed for each stimulus by comparing the maximum score on the four scales and the emotion intended to be expressed by the avatar, independently of the FACS expression level, giving a 1 if the scale with the highest score was the intended target emotion and a 0 in any other case. The proportion of concordant items for each category of stimuli (Anger, Disgust, Pain, and Fear) was then computed using, for each stimulus category, a repeated measures ANOVA on the 4 VAS scores, taking together all expression levels and using a Bonferroni correction for the *post-hoc* comparison of mean VAS scores.

#### Results

The four categories of stimuli were scored (0–100) on average higher on their respective (target) scale (Anger: *M* = 28.3, *SD* = 16.5; Disgust: *M* = 21.9, *SD* = 10.8; Pain: *M* = 31.3, *SD* = 4.3; Fear: *M* = 55.0, *SD* = 4.4) than on the other scales (see Table 2 for scores of each emotion), confirming that the dominant emotion was correctly detected in the stimulus set. However, subjects often attributed some level of Disgust to both the Anger and Pain stimuli (respectively *M* = 25.7 and *M* = 25.3), showing that Disgust was the emotion more susceptible to be misread in the stimulus set. Fear was the least ambiguous emotion; 73% of the total intensity attributed to fear stimuli loaded on the fear scores, while the accuracy scores were 40% for anger, 43% for disgust, and 40% for pain.

**Table 2:**
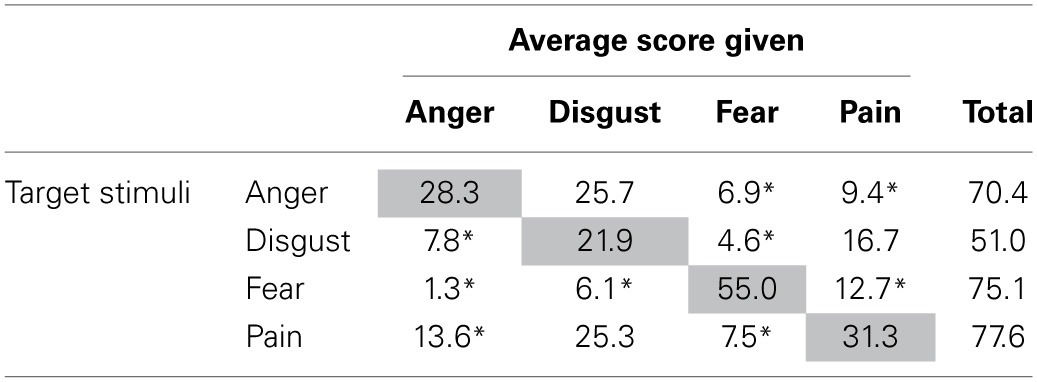
**Mean intensity scores on the 4 VAS for all 4-types of stimuli**.

*Stars mark average scores on a VAS significantly different (Bonferroni corrected) from the targeted Stimulus emotion, grayed on the same line*.

The correlations between the mean ratings of emotions provided by the participants and the targeted intensity defined as the percentage of the maximum FACS intensity (A = 20, B = 40, C = 60, D = 80, E = 100) were high and significant for all emotions (Anger: *r* = 0.77, *p* = 0.00005; Disgust: *r* = 0.88, *p* < 00001; Pain: *r* = 0.85, *p* < 00001; Fear: *r* = 0.94, *p* < 00001). The total intensity score captured some extra variance not captured by the specific intensity, as most participants scored each clip on several emotion scales rather than only one, and sometimes misattributed the dominant emotion. This led to higher correlations between the total rating scores and the targeted intensity of the facial expression (Anger: *r* = 0.88, Disgust: *r* = 0.92, Pain: *r* = 0.93, Fear: *r* = 0.90; all *p* < 0.00001).

The proportions of concordant items were very different across the four emotions, ranging from 45 to 100% (Anger: 55%, Disgust: 65%, Fear: 100%, Pain: 45%), suggesting that fear stimuli were all unequivocal, while over half of the Pain stimuli elicited another emotion (Disgust) more intensely than Pain. Overall, Fear was the clearest facial expression, being always detected when present and rarely detected when other facial expressions were displayed. In contrast, Disgust was the most confused emotion overall and it was the emotion most often incorrectly attributed to the Pain clips. Conversely, Pain was the emotion most frequently incorrectly attributed to the Disgust clips. This implies that, for experiments in which it is important to discriminate pain from other emotions, some additional tuning of the avatars will be necessary. As only one AU is common to these two emotions (see Table 1), a detailed analysis of the time-course and intensity of this AU is warranted. Moreover, one potential source of ambiguity between emotions could be related to the linearity applied to each emotion. It might be the case that the differential timing between AUs is essential for some emotions but not others.

#### Experiment 1b: pain level discrimination

In Experiment 1b, our goal was to establish the relative accuracy of the levels of pain expression as modeled from levels of the Facial Action Coding System (Ekman et al., 2002).

#### Methods

After Experiment 1a, participants were asked to complete an assessment of only the Pain clips. Thus, the same 20 pain clips (four avatars, five pain levels each) previously presented were shown again using the same procedure. This time participants were asked to rate only the level of pain they detected in each clip, following the same procedure as in the first part of the experiment, but using only one VAS. This second step in the validation procedure was undertaken because pain will be the target emotion used by EEVEE in interactive paradigms. First, a correlation between the mean pain ratings and the target pain intensity was computed. Then, we entered the Pain evaluations into a mixed-effects ANOVA with Level (5 pain intensities: A, B, C, D, and E) and Stimulus Gender (female or male) as within-subject factor, and Participant Gender (female or male) as between-subject factor. *Post-hoc* comparisons were performed with Student's *t*-test with Bonferroni correction, unilateral for Pain Levels tests, and bilateral for Gender tests.

#### Results

**Figure 6:**
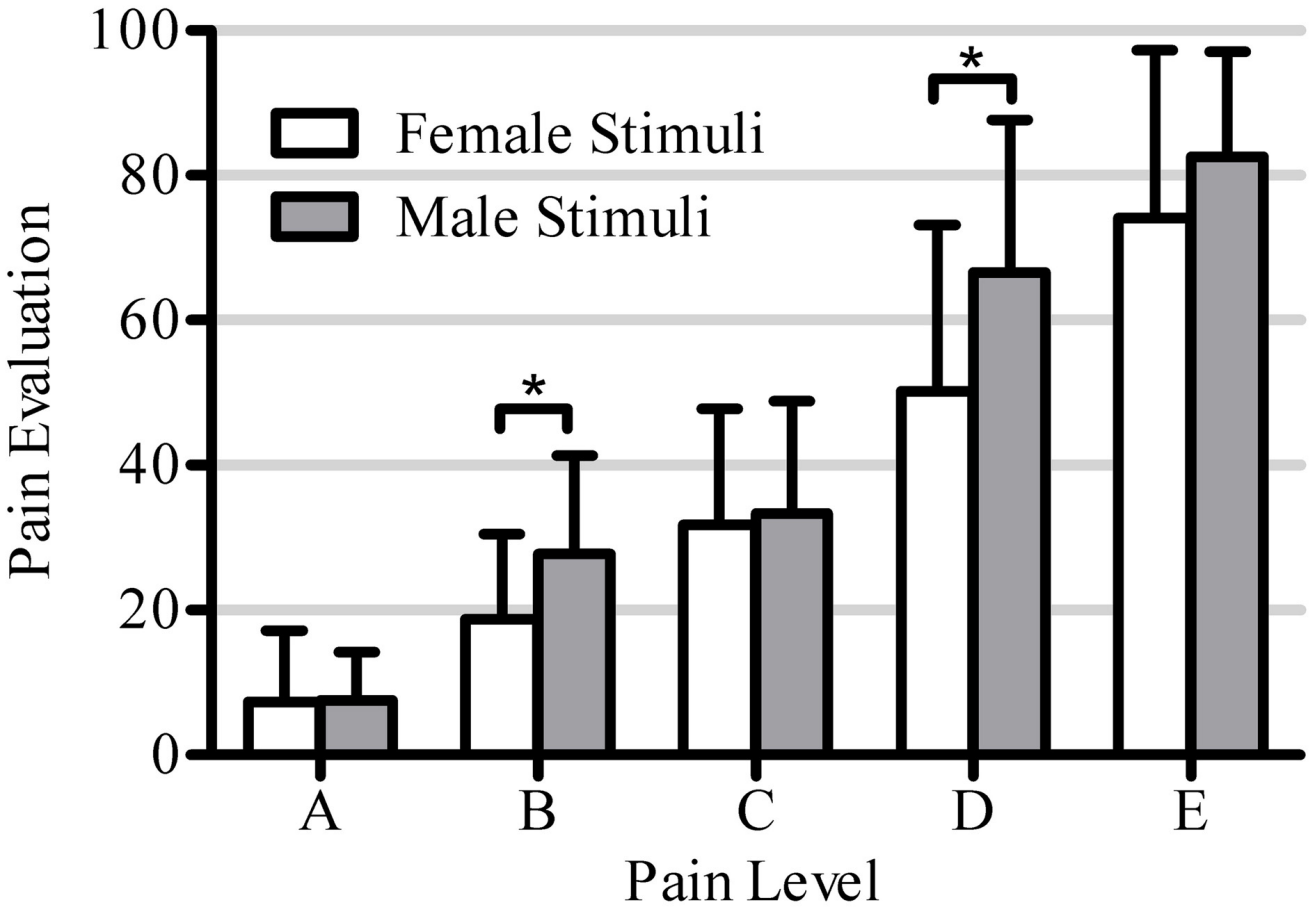
**This figure shows the significant interaction between within-subjects factors Pain Level (5 levels: A, B, C, D, E) and Stimuli Gender (2 levels: female, male) [*F*_(4, 17)_ = 5.1, *p* = 0.001].** Asterisks mark pain levels for which the male avatar stimuli receive significantly higher pain evaluation than female avatar stimuli (5 *post-hoc* tests, unilateral Bonferroni corrected *T*-tests α = 0.01).

The Three-Way ANOVA revealed a significant effect of Pain Level [A: *M* = 7.4, *SD* = 8.4; B: *M* = 23.3, *SD* = 13.5; C: *M* = 32.6, *SD* = 15.7; D: *M* = 58.5, *SD* = 23.4; E: *M* = 78.3, *SD* = 19.8; *F*_(4, 17)_ = 265.9, *p* < 0.001], and all 5 levels were statistically distinct [A vs. B: *t*_(18)_ = 21.8, *p* < 0.00001; B vs. C: *t*_(18)_ = 4.9, *p* = 0.0001; C vs. D: *t*_(18)_ = 17.8, *p* < 0.00001; D vs. E: *t*_(18)_ = 8.5, *p* < 0.00001]. No main effect of Participant Gender on the pain ratings was observed [female: *M* = 37.5, *SD* = 29.1; male: *M* = 42.7, *SD* = 31.7; *F*_(1, 17)_ = 1.4, *p* = 0.26]. There was, however, a significant main effect of Stimulus Gender showing that the pain of male avatars was rated higher than that of female avatars [female: *M* = 36.4, *SD* = 29.5; male: *M* = 43.5, *SD* = 31.0; *F*_(1, 17)_ = 15.3, *p* < 0.001], but there was no interaction between Participant Gender and Stimulus Gender [*F*_(1, 17)_ = 0.68, n.s.], or between Participant Gender and Pain Level [*F*_(1, 17)_ = 1.9, n.s.; see Figure 6].

However, the interaction between Pain Level and Stimulus Gender was significant [*F*_(4, 17)_ = 5.1, *p* = 0.001), showing that the pain of male avatars was rated significantly higher than that of female avatars at FACS levels B and D [A: *t*_(18)_ = 0.08, *p* = 0.94; B: *t*_(18)_ = 3.39, *p* = 0.003; C: *t*_(18)_ = 0.45, *p* = 0.66; D: *t*_(18)_ = 3.58, *p* = 0.002; E: *t*_(18)_ = 1.98, *p* = 0.06]. Finally, the interaction between Pain Level, Stimulus Gender and Participant Gender was not significant [*F*_(4, 17)_ = 0.59, *p* = 0.57].

#### Discussion of Experiment 1

This experiment showed that while pain intensities are correctly estimated both when pain clips are presented alone (Experiment 1b) and among clips of other emotions (Experiment 1a), participants detected on average a mixed set of emotions in all clips. Although the accuracy of emotion detection was somewhat lower than anticipated for all emotions other than fear, the ambiguity caused by the Disgust expression is consistent with what has been reported in the literature about this emotion when expressed either by humans or avatars (Noël et al., 2006; Dyck et al., 2008; Sacharin et al., 2012; Roy et al., 2013; Roy et al., unpublished manuscript). The difference between emotion detections could be related to the timing of the different action units as much as the distinct AUs. This will need further investigation for which the platform will be most useful. The study design, which proposed four rating scales at once, might have contributed to the attribution of multiple types of emotion to most clips. However, recent research suggests that human emotions are complex (not one-dimensional), and this is often reflected in facial expressions. Someone can be joyfully surprised, for instance, or angrily surprised, and not show exactly the same pattern of AUs (Du et al., 2014). This suggests that multidimensional evaluation of emotions and facial expressions could be more ecological, and reflects the fine-grained analysis needed in social interaction situations. Overall, the validation of the avatars was shown to be rather accurate but not entirely specific, suggesting that the intensities of the different AUs composing each emotion can be extracted even in the presence of extra AUs (noise). By introducing different contraction levels on different parts of a face, we may be able to accentuate the facial features that are specific to an emotion over the features that are common to multiple emotions, and attain greater specificity in future experiments. Furthermore, we could use software such as Noldus FaceReader™ to help validate the avatar expressions and refine them in an iterative fashion.

### Experiment 2

#### Objective and hypotheses

The specific objective of this second experiment was to evaluate whether different parts of the face, namely the eyes and the mouth region, have the same efficacy in communicating pain information. We expected that masking part of the facial expressions would result in observers attributing less intensity to the pain displayed in both human and avatar models. We also expected that masking the eyes would result in lower estimates of pain intensity than masking the mouth region, based on at least one study suggesting that the eyes communicate mostly the sensory component of pain while the mouth region is more associated with its affective component (Kunz et al., 2012).

#### Methods

#### Participants

A total of 36 participants (20 women) were enrolled in this experiment through emails sent to Université Laval students and personnel. Their ages ranged from 20 to 35 years old (*M* = 24.2, *SD* = 4.3). Exclusion criteria included having a neurological or psychiatric disorder, a medical condition causing pain, working in healthcare or with people suffering from pain, or having previously been enrolled in a study conducted by a member of our laboratory. The study was approved by the Research Ethics Committee of the Institut de Réadaptation en Déficience Physique de Québec. Written informed consent was obtained from all participants, who received a 10$ compensation for their participation in the study.

#### Material/Task

**Figure 7:**
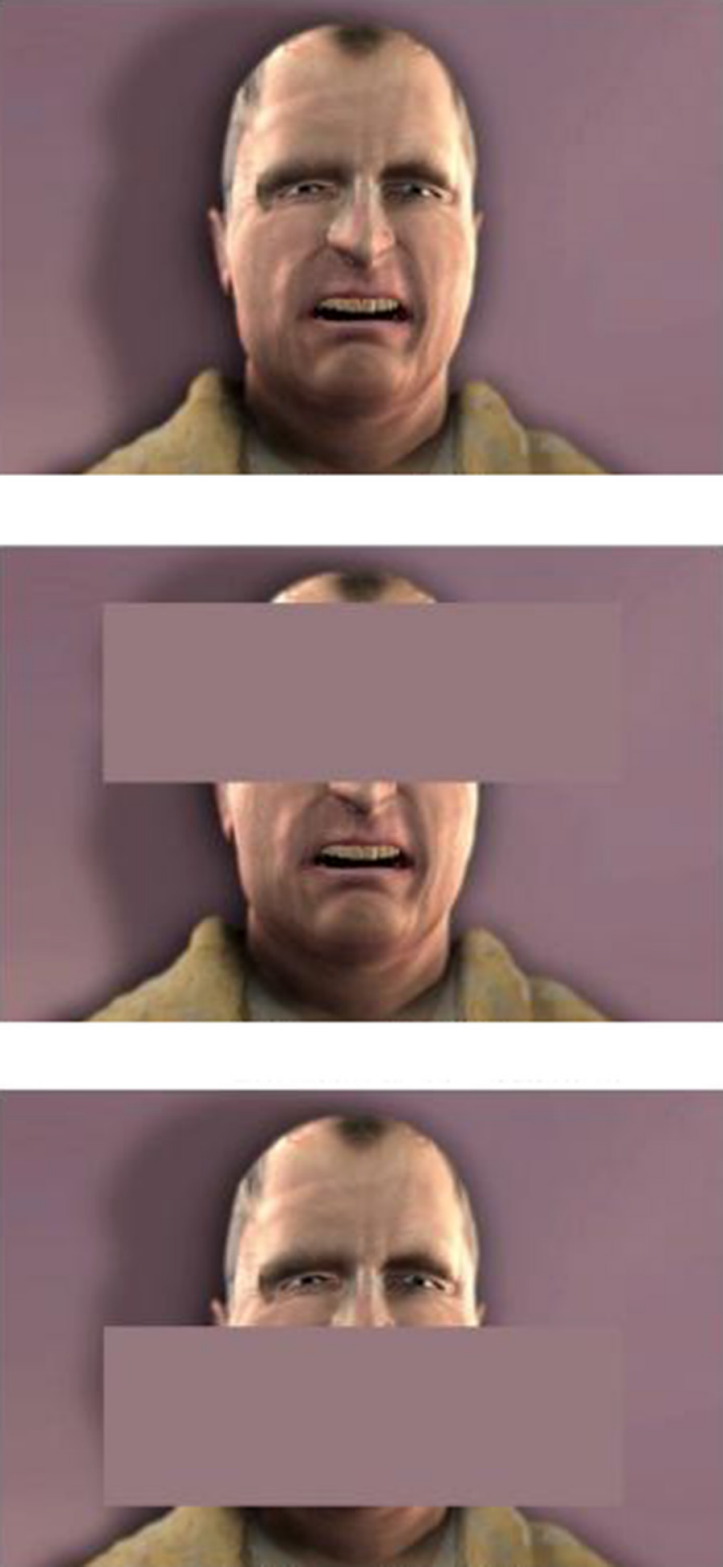
**Stimuli used in Experiment 2 showing facial expressions of pain from one of the male avatars.** All stimuli were presented in three conditions: No Mask (**top row**), Eyes Mask (**middle row**), and Mouth Mask (**bottom row**).

Human clips were taken from a validated set (Simon et al., 2008). Four models (2 males, 2 females) depicting 3 levels of pain intensity (Low, Medium, High) plus a Neutral expression, for a total of 16 different clips. Based on Experiment 1's results and previous work by others (e.g., Simon et al., 2008), Low, Medium, and High pain from the human clips were matched by 4 avatars (2 males, 2 females) depicting equivalent expressions, which corresponded to FACS levels B, C, and D respectively, plus a Neutral clip. The minimal and maximal expressions (FACS levels A and E) were not used, as they are less frequently encountered in real life, not well represented in this human data set, nor in naturalistic data sets involving patients (e.g., Lucey et al., 2011b). Thus, the experiment comprised 16 clips for each model type (Avatar, Human). This set corresponded to the Unmasked condition (see examples in Supplementary Material Videos 7–10). The Mouth Mask and Eyes mask conditions were produced by adding a static gray rectangle (mask) over the mouth or the eye regions, respectively, to each clip of the original unmasked set (see Figure 7).

#### Procedure

The four categories of clips, namely Human-Unmasked (16 clips), Human-Masked (32 clips: 16 clips with eyes masked, 16 clips with mouth masked), Avatar-Unmasked (16 clips), Avatar-Masked (32 clips: 16 clips with eyes masked, 16 clips with mouth masked) were evaluated separately in four different blocks. The block order was counterbalanced: about half the participants started with the unmasked blocks (21 out of 36 participants) while the other half started with the masked blocks. Also, the model type was counterbalanced across participants, with half of the participants starting with the human blocks and the other half starting with the avatar blocks. The order of presentation of the clips within a block was pseudo-randomized, and constrained by rules to avoid more than three successive clips of the same of gender, level, or mask within the same block. In this experiment, each clip was presented twice in each trial, with a 2-s black screen following each presentation. As with Experiment 1b, in the non-neutral clips the pain expression linearly increased in intensity for 2 s from the relaxed state (FACS neutral) to reach the maximum expression level (either Low, Medium or High pain), which was maintained for 1 s. Participants were instructed to rate the level of pain they detected in the clip, using the same procedure and pain visual analog scale (VAS) described in Experiment 1b. A Two-Way ANOVA with Model Order (2 levels: Avatars first or Humans first) and Mask Order (2 levels: non-masked or masked blocks first) as within-subjects factors was conducted to rule out order effects. A mixed-design ANOVA was conducted to rule out participant or stimulus gender effects, with Participant Gender (2) as between-subject factor and Stimulus Gender (2) as within-subject factor. To rule out possible primacy effects, a mixed design ANOVA was conducted only on the first block from each participant, with Pain Level (Low, Medium, High) as a repeated measure, and Type order (Avatars or Humans first) and Mask order (unmasked stimuli or masked stimuli first) as between subjects factors. A mixed-design ANOVA was then conducted using a Type(2)^*^Level(3)^*^Mask(3) design to compare the effect of the three types of masks (No Mask, Eyes Mask, Mouth Mask) on pain evaluations at low, medium and high pain, in avatars and humans. For this last ANOVA, *post-hoc* Student's *t*-tests were conducted to compare pain intensity ratings in the three mask conditions at each pain level, for both human and avatar models, using a Bonferroni-corrected threshold of *p* < 0.0028 (18 tests, *p* < 0.05 family-wise error), unilateral.

#### Results

First, an ANOVA on pain evaluations with Mask Order and Model Order as within subjects factors was conducted, and showed no effect of Mask Order [*F*_(1, 32)_ = 0.31, n.s.], Model Order [*F*_(1, 32)_ = 0.44, n.s.] or interaction between these 2 factors [*F*_(1, 32)_ = 1.1, n.s.]. Then a second ANOVA with Participant Gender as between subjects factor and Stimulus Gender as within subject factor showed no effect of Participant Gender [*F*_(1, 34)_ = 0.17, n.s.], but a significant effect of Stimulus Gender [female pain: *M* = 34.8, *SD* = 28.4, male pain: *M* = 38.2, *SD* = 29.5; *F*_(1, 34)_ = 56.9, *p* < 0.001]. No significant interaction was found between these two factors [*F*_(1, 34)_ = 0.1, n.s.]. The Type^*^Level^*^Mask ANOVA on the first blocks showed no effect of Mask Order [*F*_(1, 32)_ = 0.03, n.s.], Model Order [*F*_(1, 32)_ = 0.68, n.s.] or interaction between these 2 factors [*F*_(1, 32)_ = 0.3, n.s.].

The Type(2)^*^Level(3)^*^Mask(3) ANOVA yielded a significant main effect of Pain Level [Low: *M* = 30.2, *SD* = 19.3; Medium: *M* = 47.6, *SD* = 20.8; High: *M* = 63.9, *SD* = 22.8; *F*_(2, 70)_ = 380.8, *p* < 0.00001]. The main effect of Mask was not significant [No Mask: *M* = 46.5, *SD* = 25.4; Mouth Mask: *M* = 48.3, *SD* = 24.7; Eyes Mask: *M* = 47.0, *SD* = 25.2; *F*_(2, 70)_ = 1.40, n.s.], nor was the main effect of Model Type [avatars: *M* = 47.4, *SD* = 23.5; humans: *M* = 47.1, *SD* = 26.7; *F*_(1, 35)_ = 0.2, n.s.]. The interaction between Model Type and Pain Level was marginally significant [*F*_(2, 70)_ = 3.0, *p* = 0.073]. The way the stimuli were masked also interacted significantly with the type of model [Type^*^Mask: *F*_(2, 70)_ = 8.7, *p* < 0.001] and the level of pain presented [Level^*^Mask: *F*_(4, 140)_ = 3.1, *p* = 0.02]. There was also a significant three-way interaction [Type^*^Level^*^Mask: *F*_(4, 140)_ = 6.5, *p* < 0.001).

**Figure 8:**
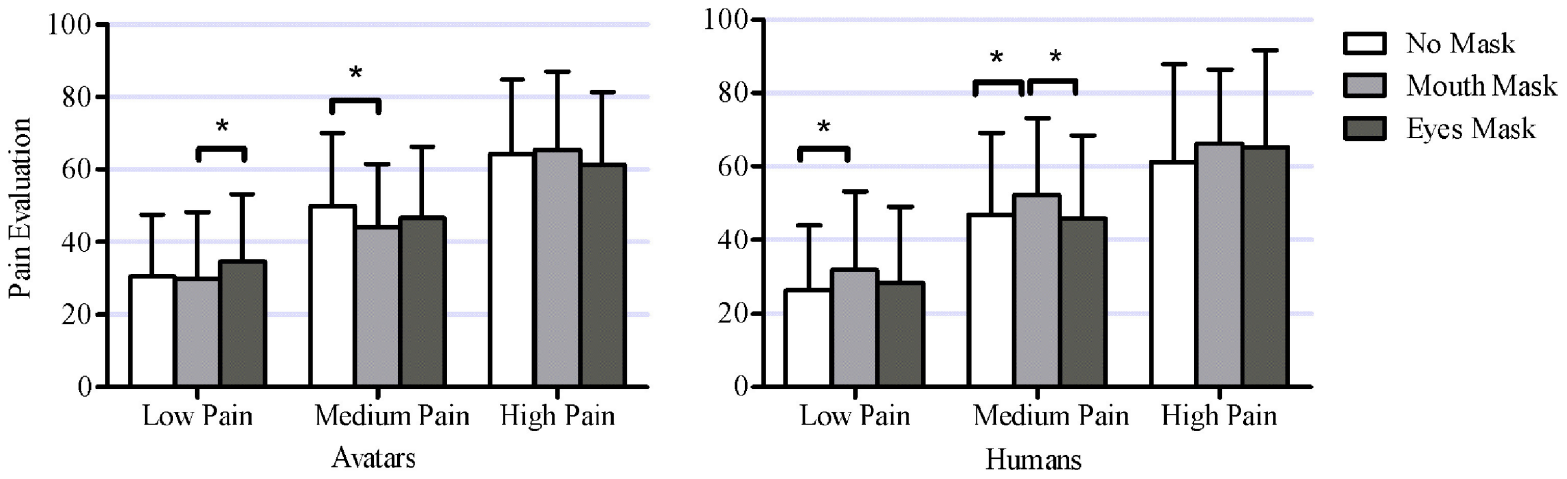
**This figure shows the significant interaction between within-subject factors Model Type (2 levels: avatars, humans), Pain Level (3 levels: low pain, medium pain, high pain) and Mask (3 levels: no mask, mouth mask, eyes mask) [*F*_(4, 140)_ = 6.41, *p* = 0.001].** Asterisks mark mask level for which evaluations at this pain level are significantly different from pain evaluation at other mask levels (18 *post-hoc* tests, with unilateral Bonferroni corrected *T*-tests α = 0.0028).

The effect of masking on pain intensity ratings depended on both the type of model and the pain level presented (see Figure 8). Because of the large number of *t*-tests performed (18), only significant differences are presented here. For avatars, intensity ratings for the Low pain level stimuli were significantly higher with the Eyes Mask than with the Mouth Mask [*t*_(35)_ = 3.28, *p* = 0.002]. For the Medium pain stimuli, intensity ratings for avatars were lower in the Mouth Mask than in the No Mask condition [*t*_(35)_ = 4.43, *p* = 0.0001]. For human stimuli, at Low and Medium pain levels, intensity ratings in the Mouth Mask condition were significantly higher than in the No Mask condition [Low pain: *t*_(35)_ = 4.45, *p* = 0.0001; Medium pain: *t*_(35)_ = 3.54, *p* = 0.001]. At Medium pain, ratings in the Mouth Mask condition were also significantly higher than in the Eyes Mask condition [*t*_(35)_ = 3.37, *p* = 0.002]. Masking did not have any significant effect on pain evaluation in the High pain condition for either avatar or human models. All other comparisons were non-significant. Thus, overall, masking the eyes or mouth region had different effects on human and avatar pain expressions; the pain evaluation in humans tended to increase when the mouth was masked, while no clear pattern emerged in avatars.

#### Discussion of experiment 2

Based on previous literature, it was expected that masking part of the facial expressions of pain would reduce intensity ratings for all stimuli, with eyes masks leading to the lowest evaluations. However, the main findings from Experiment 2 suggest a different and more complex pattern, with a significant three-way interaction between Mask, Model Type and Pain Level indicating that the effect of masking on perceived pain intensity varied according to both the type of model (avatar or human) and the level of pain displayed (low, medium, high). For high pain stimuli, masking part of the model's face (either the eyes or the mouth) had no effect on the participants' evaluations of pain intensity displayed, regardless of whether the model was an avatar or a human. For low and medium pain stimuli, while there was an effect of masking on perceived pain intensity, it was different for avatars and humans. For human stimuli, in line with our hypotheses, masking the mouth region resulted in higher intensity ratings than when the whole facial expression was perceived. For avatars however, masking the mouth led to lower perceived intensity compared to the non-masked presentation for medium pain stimuli, while no difference was found between these two conditions for low pain stimuli. Taken together, these results suggest that different facial areas could convey information best for different levels of pain. In this study, the eyes appeared to convey more intensity in human, but not in avatar models.

The differences observed between the two model types are not completely unexpected, as the facial expressions of the avatars were not based on the human models that were used in this study, but were created from the general and analytic principles of the FACS. Furthermore, the intensity was modified by changing all AUs equally, which may not be how the intensity of pain expressions varies in humans. In fact, it is likely that the different AUs involved in a given expression follow different time courses and that different emotions have different level spans for each AU, which would guide our attention to the specific aspects of emotional facial expressions. EEVEE is a valuable tool in the exploration of how fine-grained changes in facial expressions and micro-expressions can affect the communication of pain and other emotions. One caveat to note is that the human stimuli used in the current study may not be the best standard for natural facial expressions, as they show actors pretending to be in pain. While these stimuli are well validated, it will also be important to examine how the avatar stimuli created for EEVEE compare to natural facial expressions of emotions. While a set of well-validated stimuli presenting natural facial expressions of pain is available (Lucey et al., 2011b), this step may require improved modeling of facial activity in ecological facial expressions of other emotions. The finding that pain intensity ratings were significantly higher for male than for female models will need to be explored further, because even if this pattern was found in actors (Simon et al., 2006), it also seems to be inconsistent with some data suggesting that male pain is often underestimated compared to female pain (Robinson and Wise, 2003).

## General discussion

The objective of this article was to present the development of a new VR platform, the Empathy-Enhancing Virtual Evolving Environment (EEVEE) to study empathy in an interactive environment and to foster the interest for collaborative work in this domain. At this stage of development of EEVEE, we have successfully created a novel avatar architecture that can independently modulate in real time different components of its facial expression based on the FACS (Ekman et al., 2002). The first series of validation experiments showed that the avatars can produce distinct negative emotions that people can correctly identify, including pain, which is not always part of experimental emotion stimuli sets. Moreover, people readily recognized changes in intensity levels implemented through a nonlinear combination of blend shapes and animation. Not surprisingly, the more intense the emotion, the better it was identified. Note that informal debriefing about the avatars also taught us that the use of expression lines and wrinkles added realism to the avatars. Some refinements are still required, notably between disgust and other emotions, for which it will be important to conduct further experiments. Single emotion experiments can readily be launched with high confidence in the ability of avatars to express a specific emotion. For instance, the last validation experiment provided sample data on the detectability of pain faces when part of the face is covered. The first interesting finding was that the gender of the model affected pain intensity ratings in that higher ratings were provided for male than female avatars and actors. Interestingly, this finding is consistent with brain imaging data showing that male facial expressions of pain yield more activation in emotion related circuits (amygdala) than female facial expressions of pain (e.g., Simon et al., 2006) and extends it to avatars. The fact that the male and female avatars were based on the same mesh and used the same scale to modify the facial expression adds to the argument that this difference between intensity ratings of FACS-based expressions cannot be attributed to differences in the intensity of the expression *per se*, but rather points to a socio-cultural bias through which we attribute, for equivalent pain faces, more pain to male models. The main findings related to the masking procedure showed that at high pain levels, masking the eyes or the mouth does not change the accuracy of the evaluation, either for avatars or for real actors mimicking pain faces. At low and medium levels of pain the pattern was more complex; for instance at medium levels of pain, masking the eye area tended to yield lower pain ratings for avatars, but higher pain ratings for human faces, compared to the unmasked condition.

Overall, these validation experiments are encouraging and confirm the potential of EEVEE for a number of experiments in which the parametric modulation of dynamic facial expressions is essential. Future developments will include refinements in the avatars' expressions, the creation of novel avatars (of different age, gender, and ethnic background) and environments. These improvements will be based on a series of planned experiments, which will target different questions such as the influence of context (such as hospital room versus office) on the perception of an avatar's emotions. EEVEE also offers much more than new avatars as the platform is now ready to record a number of behavioral and physiological responses of the observer, synchronized with the expression of the avatars. This allows the deployment of experiments documenting the different markers that are associated with the resonance response when observing emotions in other people. Ongoing pilot work simultaneously recording heart rate, respiration rate, skin conductance, facial expressions and eye movements (via eye-tracking) in participants observing the emotional avatars will provide a first set of data to explore inter-individual similarities and differences in the physiological basis of empathy (Jauniaux et al., 2014, MEDTEQ meeting, Quebec City, October 2014).

### EEVEE in real time: proof of concept

In parallel to the refinement of the avatars, we have also begun the next development stage of EEVEE, which involves the modulation in real-time of an avatar's facial expression based on an observer's physiological responses. The current version of EEVEE can process online data recorded from a participant and modulate the facial expression of the avatar in response to it (see Video 1 for an example of changes in the avatar based on a participant's facial expression). Such a set-up can be used for instance to train people to react more (or less depending on the context) to emotional facial expressions (see part III in Future Directions).

### Future directions

In our laboratory, at least three main research themes are currently benefiting from the development of this platform: (1) pain communication and empathy, (2) the study of social cognition deficits in people with psychiatric and neurological disorders, and (3) the development of means to optimize empathy.

#### Pain communication and pain empathy

The added value of EEVEE for research on pain communication (Hadjistavropoulos et al., 2011) is immense. For instance, EEVEE will enable the systematic investigation of neurophysiological parameters of pain decoding by making it possible to manipulate and adapt the model in pain online. This will allow the identification of the most robust biomarkers of empathy for pain, and reveal different combinations of markers for different groups of individuals. EEVEE will also enable changes in the relationship between the observer and the target in pain by using specific avatars of known people (spouse, family member, friend, etc.) or people with a specific role toward the observer (physician, nurse, psychologist, etc.). We will be able to control several important variables by using avatars to compare for instance the neurophysiological and prosocial responses to seeing one's spouse compared to a stranger in pain (Grégoire et al., 2014). Finally, EEVEE will help the systematic study of inconsistencies between multiple emotional cues (e.g., voice vs. facial expression) and the detection of very low levels of expressions found in certain clinical populations (e.g., premature newborns, people with dementia), which can have a huge clinical significance.

#### Social cognition deficits

A second research axis where EEVEE could lead to important advances is the study of empathy in clinical populations showing empathy deficits, including schizophrenia, autism, personality disorders and traumatic brain injury. This axis is complementary to the first as it is based on the same models of empathy and also uses the pain of others as a model to trigger emotional responses. Initial studies have led to a number of discoveries, notably that people with high traits of psychopathy show a greater resonance response during the observation of pain (but still less empathy) than people with low traits (Marcoux et al., 2013, 2014), suggesting that they do detect pain in others. EEVEE could help determine, through controlled social scenarios, which component of empathy is specifically affected in this population. Research conducted with people having a first episode of psychosis shows that they have low to moderate levels of social cognition deficits on pencil and paper tests, which does not seem to reflect the full range of social interaction difficulties from which they typically suffer. EEVEE would help demonstrate more specifically which neuro-cognitive processes underlie these deficits and how they impact actual social interactions. EEVEE will provide several advantages, such as the possibility to introduce conflicting information in a controlled manner (for example, the avatar saying something but displaying an incongruent facial expression), to modulate affective content in a supervised environment, and to place participants in varied and complex ecologically-relevant social situations (such as familiar or less familiar, formal or informal) to study the full range of human social interactions.

#### Optimizing empathy

EEVEE can also be used as an intervention tool by exploiting the changes in a participant's physiological and neurophysiological responses to modify the avatar's facial expression, as well as their behavioral and communicative responses, so as to steer the participant toward an empathic response (a form of bio/neuro- feedback). For instance, a participant could see the avatar of his spouse, who suffers from chronic pain, displaying different levels of facial expressions of pain, which will be modulated according to a predetermined combination of neurophysiological parameters (e.g., gaze directed at the right facial features, skin conductance showing elevated affective response, etc.). The avatar would then express relief only when the participant's responses are compatible with an empathic state, but not with high levels of anxiety and distress. Verbal cues can be added periodically to add reinforcement. The same type of design could also be applied to parent-child dyads or healthcare professionals and patients. EEVEE will thus make an excellent platform to train healthcare professionals to detect and manage pain. Other groups have recently reported encouraging findings using cognitive strategies with nurses (Drwecki et al., 2011) and computerized training with physicians (Tulsky et al., 2011). In combination with neuro-stimulation techniques (see Hétu et al., 2012), EEVEE will allow for a greater range of behavioral changes, by targeting specific behaviors and triggering online stimulation to modify the cortical excitability of subjects. A simple design has shown that transcranial Direct Current Stimulation (tDCS) applied to the dorsolateral prefrontal cortex can change the way people rate static pictures of pain (Boggio et al., 2009). A better understanding of this process, with a controlled and ecological interface such as EEVEE, would allow the systematic investigation of different stimulation paradigms that can promote certain behaviors and reduce others. These examples underline the great potential of EEVEE to lead to personalized training programs complementary to cognitive approaches.

## Conclusion

EEVEE was designed as a flexible and powerful tool to study the different processes underlying human social interaction, with special emphasis on empathy. While EEVEE will help uncover the neurophysiological basis of these complex processes, it also has great potential for the study of human-machine interactions. Although considerable work has been done in the development of valid and finely modeled static and dynamic avatar facial expressions (see for example FACSGen, in Krumhuber et al., 2012), no freely accessible system, to the best of our knowledge, has combined physiological measures and facial expressions of avatars into a dynamic social interactive tool. The addition of new inputs (e.g., postural analysis and speech recognition) and outputs (speech production), as well as the implementation of machine learning algorithms on the large quantity of data that will be generated, will bring EEVEE to the next level. Already, EEVEE is a unique platform for studying empathy in a number of populations suffering from neurological and psychiatric disorders. Currently, the platform allows the production of different levels of facial expressions of the following emotions for four different avatars (2 males, 2 females): Pain, Anger, Disgust, Fear, Joy, Surprise, Sadness. These avatars can currently be shared with the scientific community as separate video clips by contacting the corresponding author. Once the complete platform is compiled in an executable format, it will be made available through the corresponding author's website. Making this platform available to the scientific community is a priority for our team as this will propel its development and the likelihood that it will contribute directly to improving social interactions in humans, which in turn can improve the quality of life of many different clinical populations.

### Conflict of interest statement

The authors declare that the research was conducted in the absence of any commercial or financial relationships that could be construed as a potential conflict of interest.

## Supplementary material

Demo Video 1 demonstrates the interactions between an avatar in the EEVEE platform and the FaceReader™ software. The avatar mirrors the pain action units (AU 6, 7, 9, 10, and 43) of the participant as detected by FaceReader™. As described in Section Pain Facial Actions Units, the blendshape's value increase linearly depending of the FACS' intensity and the blendshape's value is interpolated over time.

Examples of emotions This video set illustrates the different emotions used in Experiment 1 (anger, disgust, fear, pain, plus neutral) expressed by one of the avatars at level D (80% of maximum Action Units intensity): Video 2 = Anger Level D; Video 3 = Disgust LevelD; Video 4 = Fear LevelD; Video 5 = Pain LevelD. Video 6 = Neutral.

Pain levels This video set illustrates different levels of pain (B = 40%, C = 60%, D = 80% of maximum Action Units intensities) used in Experiment 2 and expressed by one avatar: Video 7 = Pain LevelB; Video 8 = Pain LevelC; Video 9 = Pain LevelD; Video 10 = Neutral.
